# Silencing and Un-silencing of Tetracycline-Controlled Genes in Neurons

**DOI:** 10.1371/journal.pone.0000533

**Published:** 2007-06-20

**Authors:** Peixin Zhu, M. Isabel Aller, Udo Baron, Sidney Cambridge, Melanie Bausen, Jan Herb, Jürgen Sawinski, Ali Cetin, Pavel Osten, Mark L. Nelson, Sebastian Kügler, Peter H. Seeburg, Rolf Sprengel, Mazahir T. Hasan

**Affiliations:** 1 Max Planck Institute for Medical Research, Heidelberg, Germany; 2 Max Planck Institute of Neurobiology, Munich-Martinsried, Germany; 3 Epiontis GmbH, Berlin, Germany; 4 Department of Clinical Neurobiology, University of Heidelberg, Heidelberg, Germany; 5 Paratek Pharmaceuticals Inc., Boston, Massachusetts, United States of America; 6 Department of Neurology, University of Göttingen Medical School, Göttingen, Germany; The Babraham Institute, United Kingdom

## Abstract

To identify the underlying reason for the controversial performance of tetracycline (Tet)-controlled regulated gene expression in mammalian neurons, we investigated each of the three components that comprise the Tet inducible systems, namely tetracyclines as inducers, tetracycline-transactivator (tTA) and reverse tTA (rtTA), and tTA-responsive promoters (P_tets_). We have discovered that stably integrated P_tet_ becomes functionally silenced in the majority of neurons when it is inactive during development. P_tet_ silencing can be avoided when it is either not integrated in the genome or stably-integrated with basal activity. Moreover, long-term, high transactivator levels in neurons can often overcome integration-induced P_tet_ gene silencing, possibly by inducing promoter accessibility.

## Introduction

**Figure 1:**
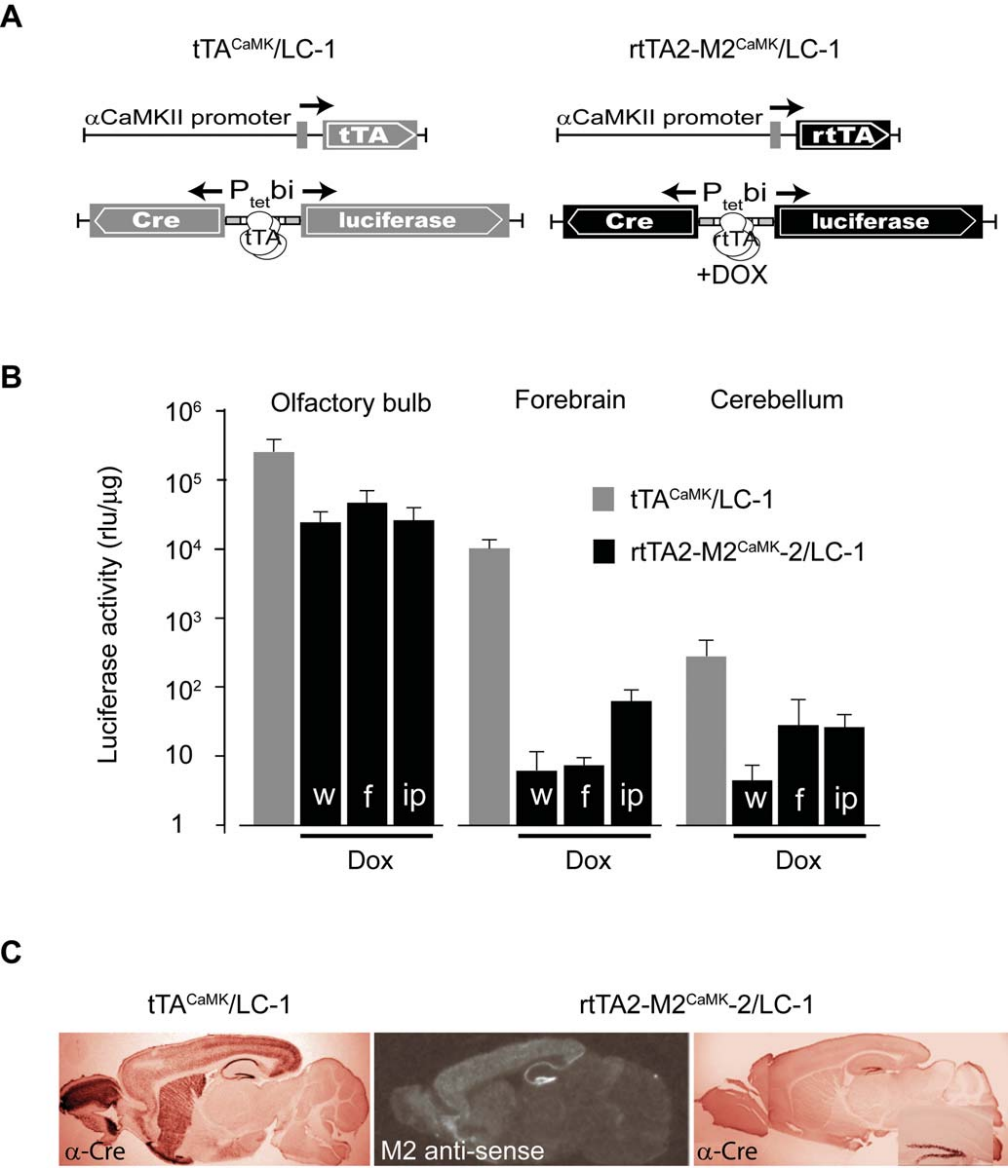
Functional brain specific rtTA mice and tetracycline-induced, rtTA-dependent gene activation in the forebrain. (A) Schematics depicting the double-positive mice harboring constructs of tTA (left) and rtTA (right) under the control of the αCaMKII promoter and responder genetic modules under control of bidirectional Tet promoter (P_tet_bi) to express Cre recombinase and the firefly luciferase genes. Notice that the tTA and rtTA activate P_tet_bi in the absence and presence of Dox, respectively. (B) Luciferase activity in different brain subregions in double-postive transgenic mice (rtTA2-M2^CaMK^-2×LC-1) treated with Dox by different delivery routes (black bars, w (water), f (food) and ip (intraperitoneal injection). For comparison, luciferase activity in double transgenic mice (tTA^CaMK^×LC-1) is also shown (grey bars). Luciferase activity presented on a log scale. (C) Forebrain-specific expression of Cre recombinase in tTA^CaMK^/LC-1 mice (left). Specific detection of rtTA2-M2 RNA in brain slices of rtTA2-M2^CaMK^-2 mouse. Gene activation induced for two weeks of 9TB-Dox treatment and Cre signal is visualized by immunohistochemistry (right, insert shows a magnified image of dentate gyrus).

Since the first description of the bacterially-derived Tet-controlled inducible systems [1]–[3], gene activation and inactivation has been achieved in different cell types in animals. The tetracycline-controlled transactivator-responsive minimal Tet promoters (P_tet_/P_tet_bi) [1], [4] are activated by the tetracycline transactivator (tTA) [1] (Fig. 1A-left) and inactivated by tetracycline (Tet-off) or its derivatives such as doxycycline (Dox). The reverse tTA (rtTA) is a complementary genetic module uniquely suited for rapid gene activation by addition of Dox (Tet-on) (Fig. 1A-right) in cultured cells [1] and *in vivo* [5], [6]. Both systems have been successfully employed in transgenic mice for studying various biological functions [7].

As of today, the Tet systems are unique for reversible control of gene expression in higher eukaryotes [1]–[3]. Switching gene expression “on” and “off” is of extreme importance in understanding the function of genes in phenotypes. This becomes especially important when studying gene function(s) in the adult nervous system. Key studies provided the first strong evidence that the Tet-regulated gene expression can be used to analyze the involvement of genes in cognition in the mouse [8]. The successful employment of the α-subunit of CaMKII promoter for regulating gene expression in the forebrain was first demonstrated with the Tet-off system [9] and later with the Tet-on system [10], [11].

However, in some studies in the central nervous system (CNS) employing the Tet-off system, the full reactivation of Tet-regulated genes after Dox withdrawal was difficult to achieve once their expression had been suppressed prenatally by Dox [12]–[14]. As a consequence, reactivation was slow, expression patterns changed and expression levels failed to reach their original maximal values [12]–[14]. Other studies observed similar difficulties with the rtTA-dependent gene activation in the mouse brain [15]. Possible reasons include poor Dox penetrance across the blood brain barrier (BBB) [15], weak P_tet_/P_tet_bi activation in adult mice [16], [17] and P_tet_/P_tet_bi susceptibility to silencing [18]–[20].

Here we investigated the regulation of several Tet-responder genes integrated at different sites in the genome [21]–[23] and in an episomal state [24]–[27] in the mouse. To regulate the Tet-responder genes, we employed transgenic mice with both forebrain-specific tTA [9] and rtTA expression (present study) and monitored responder gene activity by enzymatic activity and immunohistological analyses of brain slices. We have discovered that stably-integrated P_tet_bi modules in all responder mice become functionally silenced in the majority of neurons if P_tet_bi remain inactive during development whereas long-term, high transactivator levels in neurons can overcome P_tet_bi gene silencing. We also found that basal P_tet_/P_tet_bi activity yields permissive conditions for faster activation/re-activation, possibly by providing better accessibility of P_tet_/P_tet_bi to tTA/rtTA.

## Results

### Forebrain-specific rtTA expression in transgenic mice can activate Tet-induced gene expression in some neurons

For controlling transgenic Tet-responder genes, we used activator mice which express tTA [1], [9] and rtTA-M2 [2], [28] from the promoter for αCaMKII [9], which is active mainly in principle forebrain neurons. We engineered novel tTA and rtTA variants by replacing the potent activation domain, VP16, with three minimal activation domains of about 14 amino acids each, abbreviated as tTA2 and rtTA2-M2 [28]. Next, we generated five rtTA2-M2^CaMK^ mouse lines. For measuring gene activation, we employed LC-1 reporter mice [21], which harbor a bidirectional Tet-promoter cassette (P_tet_bi) with genes for luciferase (Luc) and Cre recombinase (Cre) (Fig. 1A). We treated five rtTA2-M2^CaMK^ lines crossed to LC-1 with Dox and gene activation was visualized by both luciferase activity measurements from different brain areas and Cre immunohistochemistry on fixed brain slices. Robust transactivation (luciferase activity) was apparent in the olfactory bulb and low transactivation in the forebrain by three routes of Dox delivery; water, food and intraperitoneal injection (i.p.) (Fig. 1B and data not shown). In none of our five rtTA2-M2^CaMK^ lines could we detect the expected robust forebrain specific gene activation observed with the tTA system (tTA^CaMK^/LC-1 mice) [9], [14], [21] (Fig. 1B and data not shown), despite the fact that rtTA2 mRNA was clearly detectable throughout the forebrain, with dentate gyrus (DG) granule cells having the highest rtTA2 mRNA levels in all rtTA2-M2^CaMK^ lines tested (Fig. 1C-middle and Fig. S1A). One possible explanation for these results was lower effectiveness of Dox for rtTA-dependent gene activation.

To find a better substitute for Dox, we used a more hydrophobic Dox derivative, 9-tert-butyl-doxycycline (9TB-Dox), which when compared to Dox has approximately 10-times higher binding affinity to the tet repressor (compound available from Mark Nelson, Paratek Pharmaceutical, Boston, USA, data not shown) and thus it is also approximately 10-times more effective than Dox in rtTA2-M2 dependent gene activation in HeLa cells (data not shown). We found that granule cells of DG, the subregion with the highest rtTA2 mRNA level (Fig. 1C-middle and Fig. S1A), showed the best induced Cre expression in all rtTA2-M2^CaMK^ lines tested, but only a few Cre positive nuclei were seen in other forebrain regions (Fig. S1B; see Dox-induction Protocol 1 in Materials and Methods). The influence of high rtTA2 mRNA levels for P_tet_bi gene activation in different brain regions is clearly apparent in the striatum of rtTA2-M2^CaMK^-4 mice (Fig. S1A, B). Increasing the treatment time with 9TB-Dox facilitated rtTA2-M2 dependent gene activation (rtTA2-M2^CaMK^-2 mice) in a larger fraction of DG granule cells when tested with the responder mouse line, LC-1 (Fig. 1C-far right; see Protocol 2 in Materials and Methods), but poorly in the hippocampal CA1 and CA3 fields and in cortex, raising concerns that the BBB might be impeding Dox availability in the mouse brain.

### Dox delivery is not the only limiting factor for lack of rtTA-dependent gene activation

**Figure 2:**
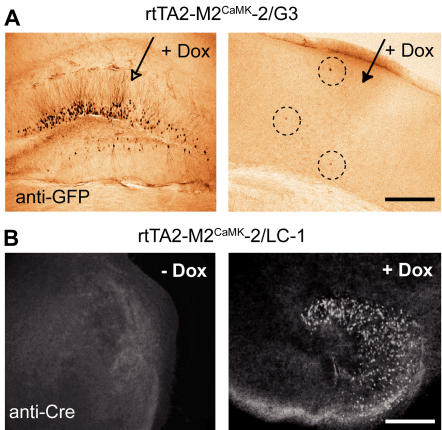
Direct Dox delivery to brain tissues. (A) Induced gene expression in the rtTA2-M2^CaMK^-2/G3 mouse by direct Dox injection into brain regions in vivo, DG granule cells (left) and cortex (right). (B) Gene expression in organotypic slices of rtTA2-M2^CaMK^-2/LC-1 mice either without Dox (left) or treated with Dox (right). Scale bars, 200 µm.

To investigate whether the BBB was the reason for inefficient Tet-controlled gene activation in hippocampus and cortex of our rtTA2-M2^CaMK^-2 mice, we used the transgenic line G3 which harbors under P_tet_bi control the LacZ and GFP genes [22], injected into the different brain regions 10 µg of Dox in 1 µl (0.7% NaCl) and tested for reporter gene activation. Seven days after Dox injection, GFP expression was induced mainly in DG granule cells on the injected side of the brain (Fig. 2A), but only in a few cortical neurons. These results demonstrate that P_tet_bi activation in G3 responder mice failed even when Dox availability was not a limiting factor.

Our result also finds support from Dox-treatment of organotypic hippocampal slices derived from rtTA2-M2^CaMK^ -2/LC-1 mice. We again used the P_tet_bi–directed Cre expression of the responder LC-1 to exclude line specific effects. Dox added to the culture medium activated P_tet_bi expression only in DG granule cells but again not in CA1/CA3 neurons (Fig. 2B). P_tet_bi activation was not detected without Dox. These results suggest that the responder genes controlled by P_tet_bi may become silenced in neurons during development.

### For P_tet_bi re-activation, incomplete suppression of tTA-dependent gene expression is necessary and sufficient

**Figure 3:**
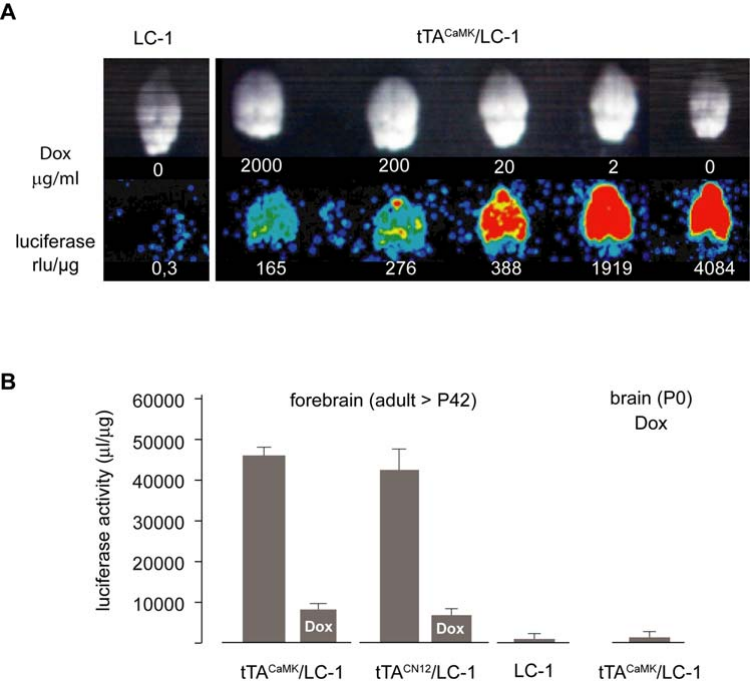
Incomplete gene suppression by Dox. (A) Dox-controlled, tTA-dependent gene inactivation of luciferase activity detected in intact brain and lysates from double-positive transgenic mice (tTA^CaMK^×LC-1), either untreated or treated with different Dox concentrations in the drinking water for three weeks. Single-positive transgenic LC-1 mouse brain also shown as a control (far left). (B) Two different strains of double-positive mice, (tTA^CaMK^×LC-1 and tTA^CN12^×LC-1), were used to measure luciferase activity in the forebrain (displayed on a linear scale), either untreated or Dox-treated (2 mg/ml of Dox in the drinking water) for three weeks. Also, luciferase activity was measured from the forebrain in newborn embryos treated with Dox throughout development (tTA^CaMK^×LC-1, far right). Scale bar, 100 µm.

We described previously reversible P_tet_bi regulation in neurons in the brains of tTA^CaMK^/LC-1 mice [21]. Such a reversible P_tet_bi regulation should not be possible if transcriptionally inactive P_tet_bi become silenced. Therefore, we analyzed in the brains of tTA^CaMK^/LC-1 [21] mice the transcriptional activity of P_tet_bi after switch-off by Dox treatment. For a detailed examination we treated adult mice (P60) with different Dox concentrations (2 to 2000 µg/ml in drinking water) and measured down-regulation of P_tet_bi-controlled luciferase expression by bioluminescence imaging of brain explants and biochemical measurements of whole brain extracts (Fig. 3A). Increasing Dox concentrations down-regulated Luc activity in a graded manner. High Dox concentration (2 mg/ml) reduced Luc activity by approximately 95% but not to background levels observed in LC-1 mice not carrying the αCaMKII promoter tTA transgene (Fig. 3A). To bypass the BBB, we treated throughout gestation newborn tTA^CaMK^/LC-1 mice with 2 mg/ml of Dox in the drinking water (Fig. 3B) when BBB does not exist and Dox availability into brain tissue is not a limiting factor and found complete suppression of gene activity in the brain of newborn pups. To rule out line specific effects, we also employed a different αCaMKII promoter tTA line, CN12 [29], and again found incomplete gene inactivation by Dox (2 mg/ml, 3 weeks) (Fig. 3B). These results indicate that a Dox concentration needed for complete P_tet_bi suppression cannot be reached in the brain by including 2 mg/ml of Dox in the drinking water. Since complete suppression of tTA-dependent gene activity in cultured mammalian cells [1] requires 10 ng/ml of Dox, it seems plausible that the effective Dox concentration in brain tissue *in vivo* will not exceed 10 ng/ml.

Our results suggest that persistent basal P_tet_bi activity prevents the P_tet_bi locus from being silenced. Transcriptional activity might keep the chromatin locus “open”, thus providing permissive conditions for gene re-activation upon removal of Dox.

### Episomal P_tet_bi responders are activatable in rtTA2-M2^CaMK^-2 mice

**Figure 4:**
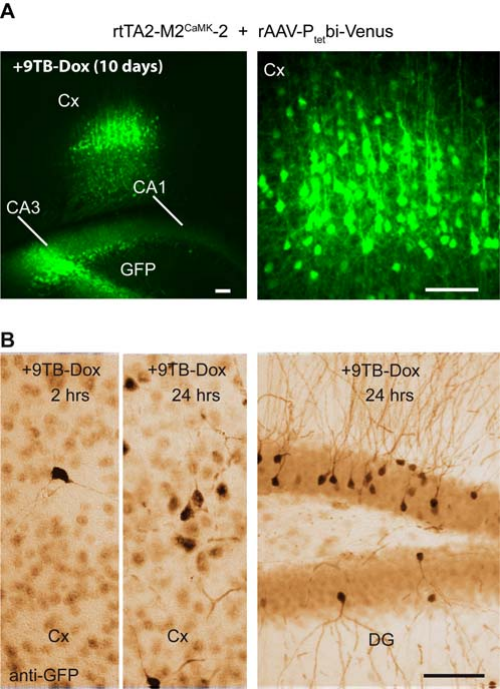
Episomal P_tet_bi responder gene activation in rtTA2-M2^CaMK^-2 mice. (A) rAAV-P_tet_bi-GFP^venus^/Cre infected into the brain of rtTA2-M2^CaMK^-2 mice and robust P_tet_bi gene activation (live GFP fluorescence) was clearly apparent throughout the infected sites (cortex, Cx, and hippocampal layers CA3 and CA1) after 10 days of treatment with 9TB-Dox. (B) Rapid gene activation is also apparent within hours of 9TB-Dox treatment. Scale bars, 100 µm.

To provide direct proof that chromosomally-inserted P_tet_bi become transcriptionally inaccessible for functional tTA/rtTA dependent gene expression, we delivered by stereotactic injection recombinant adeno-associated virus (rAAV) carrying the P_tet_bi responder genes Cre and GFP into the hippocampus and cortex of rtTA2-M2^CaMK^-2 mice. As AAV remains in an episomal state [24], [25], we asked whether that non-integrated state might provide a permissive condition for P_tet_bi, possibly by increasing promoter accessibility [30]. Indeed, we detected in the cortex and hippocampus of rtTA2-M2^CaMK^-2 mouse brains after viral injection with rAAV-P_tet_bi-GFP/Cre robust GFP expression by 9TB-Dox (i.p; 1.5 mg/injection, every 48 hours, 10 days). A large number of cortical neurons were visible by live GFP fluorescence and in fixed brain slices after 10 days of 9TB-Dox treatment (Fig. 4A, see Protocol 2 in Materials and Methods). Mice not treated with 9TB-Dox did not show P_tet_bi activation (data not shown). Because non-integrated rAAV-P_tet_bi-GFP/Cre, without tTA, have basal promoter activity, we used low virus titers to reduce background signal (data not shown). We estimate that 9TB-Dox induced, rtTA2-M2 dependent gene activation of rAAV-P_tet_bi-GFP/Cre can be as high as 20-fold, as determined by two-photon imaging of GFP fluorescence in fixed brain slices (data not shown). Further, we find that gene activation occurred rapidly within hours after a single pulse of 9TB-Dox by i.p. injection (Fig. 4B).

These results reveal two key points: first, episomal rAAV vectors are highly permissive for gene activation in neurons *in vivo*. Second, neuronal rtTA2-M2 levels in rtTA2-M2^CaMK^-2 mice are functionally sufficient for rAAV-P_tet_bi activation. Therefore, it seems most reasonable to conclude that chromosomally-integrated P_tet_bi modules are functionally silenced in neurons.

### Prolonged high tTA expression overcomes P_tet_bi silencing in neurons

**Figure 5:**
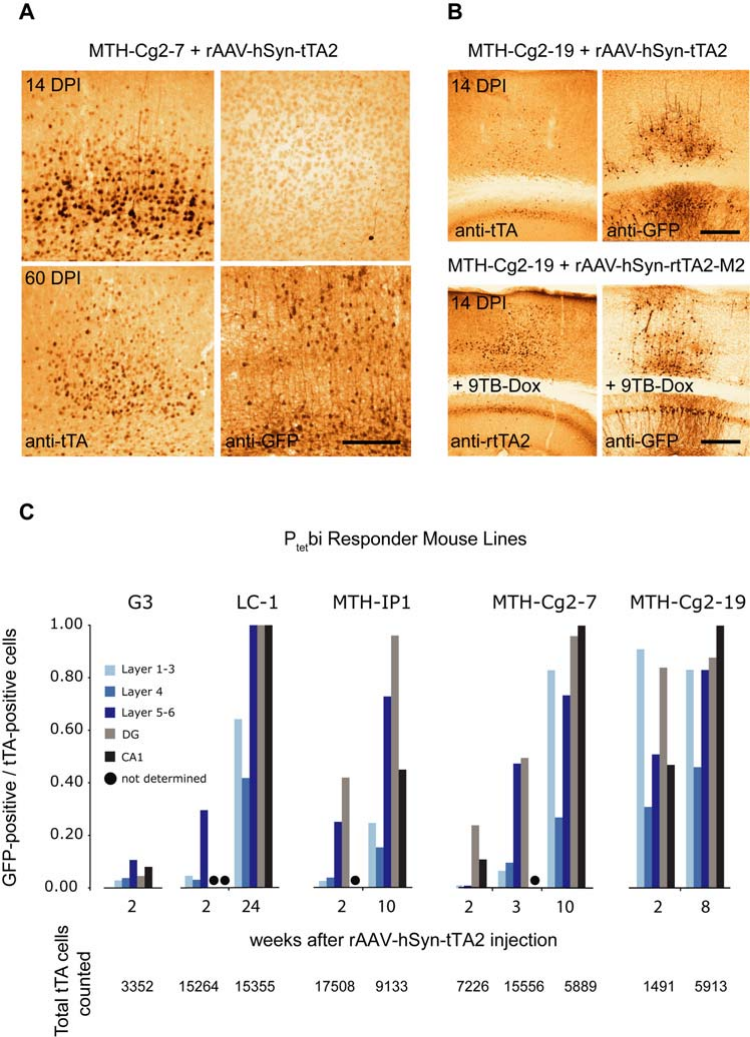
Un-silencing of P_tet_bi in neurons. (A) rAAV mediated high tTA expression in cortical neurons of responder mice (MTH-Cg2-7) after two weeks (upper panel) and eight weeks (lower panel). (B) MTH-Cg2-19 mice with “basal” P_tet_bi activity infected either with tTA2 (upper panel) or with rtTA2-M2 (lower panel). Serial brain sections were stained for tTA/rtTA2-M2 (left panel) and induced GFP expression (right panel). (C) Fraction of GFP-positive/tTA-positive neurons in different brain subregions (cortical layers, CA1 and DG) of different responder lines over time. Day post infection (DPI). Scale bars, 250 µm.

It is well known that the potent transcriptional activator derived from Herpes Simplex virus, VP16, has anti-silencing gene activity [30]–[32]. Since tTA2 [28] is equipped with three minimal activation domains with similar properties, we investigated whether strong tTA expression in neurons of the responder mice would overcome P_tet_bi silencing. Therefore, we delivered either tTA2 or rtTA2-M2 under control of the human synapsin promoter (hSyn) by injecting rAAV-hSyn-tTA2 into cortex and hippocampus of Tet-responder line MTH-Cg2-7 that showed robust P_tet_bi promoter activity in the genetic background of tTA^CaMK^ mice (Fig. S2A). Two weeks after rAAV infection of MTH-Cg2-7, tTA2 expression was apparent in cortical neurons but P_tet_bi activation was observed in only a very few neurons as revealed by GFP immunostaining (Fig. 5A-upper right panel and Fig. 5C). Similar results were found for eight different “tight” Tet-responder mouse lines. In total we analyzed approximately 100,000 neurons on serial sections (6 slices per mouse and 2 mice/line) immunostained for GFP and tTA2. For eight different “tight” Tet-responders, we found that after two weeks of virus infection, GFP gene activation was apparent in only a small fraction of neurons in CA1, DG and cortex, whereas after three weeks and beyond GFP activation was nearly complete in CA1 and DG, but remained incomplete in cortical neurons (Fig. 5C and data not shown). Next, we wanted to determine whether long-term expression of tTA2 would overcome P_tet_bi silencing. Consistent with the role of the transcriptional activation domain of VP16 in gene un-silencing [30]–[32], we found that high tTA2 levels in neurons gradually increase P_tet_bi activation over time and thus P_tet_bi un-silencing (Fig. 5A-lower panel and C). However, in mouse line MTH-Cg2-19 with basal P_tet_bi activity [23] we observed widespread GFP expression in CA1, DG and cortical subregions already after two weeks of viral tTA2 expression (Fig. 5B-upper panel), and 9TB-Dox induced gene activation in MTH-Cg2-19 mice was also apparent to similar extend when rtTA2-M2 was delivered by rAAV (Fig. 5B-lower panel). These results indicate a more permissive nature of P_tet_bi in the MTH-Cg2-19 line (Fig. 5B). Again, GFP expression was incomplete in a majority of cortical neurons (∼40%) with lowest response observed in cortical layer 4 (Fig. 5C). It is conceivable that different neuronal populations differ in their requirements for gene re-activation of silenced P_tet_-controlled genes.

Although viral-mediated tTA/rtTA delivery into neurons of MTH-Cg2-19 mice identifies this line as “more permissive” than the other eight responder lines tested so far, 9-TB-Dox induced gene activation in MTH-Cg2-19/rtTA2-M2^CaMK^-2 double-positive mice remained restricted to DG granule cells (Fig. S3A). It is possible that basal P_tet_bi gene activity in neurons of MTH-Cg2-19 mice is still insufficient and rtTA-M2 levels provided by rtTA2-M2^CaMK^-2 mice are not high enough for un-silencing. As a control, we can show that MTH-Cg2-19 mice can be activated in a forebrain-specific manner in crosses with tTA^CaMK^ mice (Fig. S3B, also see Fig. S2B).

Altogether, we have demonstrated here that prolonged high tTA2 expression in responder mice can induce P_tet_bi re-activation, possibly due to the counter-silencing effects of the transcriptional activator [30]–[32].

## Discussion

Over the last 10 years, the reported performance of the tTA system for reversible control of gene expression in neurons has been consistently remarkable. For example, an activator line driving tTA expression in principle neurons under the control of the αCaMKII promoter, tTA^CaMK^ [9], has been elegantly used to reversibly regulate the expression of various responder transgenes for studying synaptic plasticity and learning and memory [9], [12]. Targeted expression of the tTA protein specifically in CA1 principle neurons has further revealed a direct link between selective neuronal circuit function(s) and memory consolidation [33]. On the other hand, in spite of a few reported successes [10], [11], [34], others report difficulties with the Dox-induced, rtTA-dependent gene expression in neurons [15], [16], [18]. The controversial performance of the rtTA system in neurons prompted us to systematically examine individual components of the Tet system, namely Dox availability across the BBB, rtTA levels and P_tet_/P_tet_bi activation.

We have clearly demonstrated here that lack of gene activation by rtTA in neurons in adult mice is mainly due to the silencing of stably-integrated P_tet_/P_tet_bi modules. By combining rtTA2-M2 and an improved Dox derivative, 9TB-Dox, we have provided strong evidence for fast and robust gene activation in neurons when episomal P_tet_bi modules had been introduced into the brain of our rtTA2-M2^CaMK^-2 mice (live GFP fluorescence, Fig. 4A). Consistently, we could not achieve gene activation in eight different “tight” Tet-responder lines tested with tTA2 delivered via rAAV *in vivo* into brain areas (Fig.5A-upper panel, Fig. 5C and data not shown). This indicates that the poor activation of P_tet_bi responder genes in the mouse brain is neither due to the poor penetration of Dox across the BBB nor to low rtTA levels in neurons, but is most likely due to silencing of the stably-integrated P_tet_bi in the genome.

We asked whether there are specific environmental conditions which would prevent the P_tet_bi locus from becoming silenced. Towards this question, we found that unlike “tight” responders where P_tet_bi modules integrated in a genomic site with no intrinsic activity, P_tet_bi integrated in a genomic site with basal P_tet_bi activity will keep the locus permissive. We were able to identify one such mouse line, MTH-Cg2-19 [23], which we label as “permissive line” because tTA-dependent P_tet_bi activation in these mice occur earlier compared to “tight” lines (compared Fig. 5A-upper panel with Fig. 5B-upper and lower panels, also see Fig. 5C). These observations strongly support the hypothesis that basal P_tet_bi activity can keep the chromatin locus accessible for tTA/rtTA dependent gene activation.

Consistent with this idea, we have also discovered that basal P_tet_bi activity is essential for achieving multiple cycles of gene inactivation by Dox and re-activation upon Dox withdrawal in tTA-activator/P_tet_bi-responder mice [9], [21], [35]. Previously, we showed that tTA-dependent gene expression in tTA^CaMK^/LC-1 mice begins prenatally, at embryonic day 12.5 (E12.5) [14]. It is possible that early tTA expression in neurons during development activates P_tet_bi and antagonizes silencing. Moreover, even when tTA^CaMK^/LC-1 and tTA^CN12^/LC-1 mice are kept on Dox (2 mg/ml) for weeks, luciferase activity is not completely suppressed to levels observed in single-positive LC-1 mice (Fig. 3A, B), rendering the P_tet_bi locus permissive in neurons and enabling gene re-activation upon Dox removal [9], [21].

How would one create conditions to unlock the silencing in “tight” Tet-responder lines? We found that even high tTA2 expression can not activate P_tet_bi in neurons two weeks after rAAV infection but long-term (three weeks and beyond) high tTA2 levels in neurons can overcome P_tet_bi gene silencing (Fig. 5A-lower panel, C), perhaps involving chromatin remodeling by transcriptional activation domain in tTA/rtTA modules [28], [36]. We have also found that tTA2-dependent P_tet_bi un-silencing is nearly complete in neurons of hippocampus, particularly in the DG subregion but incomplete in the cortex (Fig. 5C). Why about 40% of cortical neurons remain resistant to gene activation even in the presence of high tTA2 levels is not clear. It is conceivable that diverse neuronal types might have different requirements for gene activation, silencing and un-silencing (or re-activation). In our studies, gene activation appears permissive in olfactory receptor neurons and DG granule cells, consistent with previous observations [37]. These cell types might be generally more resistant to gene silencing, possibly because they regenerate throughout life.

To reveal molecular changes which might orchestrate P_tet_/P_tet_bi silencing, we performed DNA methylation analyses on P_tet_bi, but were unable to correlate the observed fraction of DNA methylation of genome-integrated P_tet_bi with the fraction of P_tet_bi silencing in neurons. We also failed to find a significant difference in DNA methylation in brain and liver tissues (data not shown). Individual DNA sequencing of clones of P_tet_bi from brain tissue revealed that only 4% of the clones show strong methylation over the entire sequence of P_tet_bi (Fig. S4, also see “Notes on P_tet_bi methylation”, Fig. S5). This result does not account for about 100% of cortical neurons (representing approx. 60% of total DNA pool) for which we observe strong functional P_tet_bi silencing (Fig. 1C-far right and Fig. S1B, Fig. 5A-upper panel and 5C). The simple fact that we have identified a strongly methylated P_tet_bi in cortical genomic DNA hints at epigenetic control mechanisms in certain cell types in the brain. Therefore, we conclude that for P_tet_bi silencing other mechanisms, besides DNA methylation, are likely to be more relevant, such as chromatin-controlled promoter accessibility to transcriptional factors [30].

We thus favor the idea that different cell types might have different degrees of accessibility to P_tet_/P_tet_bi [30]. What determines whether a gene is either silenced permanently or is allowed to un-silence by an environmental signal? Of special interest is the ability of immediate-early-genes (IEGs), including c-fos and arc, to become transcriptionally activated by a specific stimulus [38]. It is well known that IEGs have different levels of basal transcriptional activity in different cell types in the “uninduced state” [38]. It is still unclear whether basal transcriptional activity of IEGs provide a permissive condition for gene activation in certain neuron types. It is tempting to speculate that basal IEG activity might be needed for activity-induced rapid gene activation in neurons.

Our studies have delineated the necessary conditions for reversible control of gene expression in neurons. First, we have shown that the stably-integrated P_tet_bi becomes silenced, provided it is inactive during development, and that high tTA/rtTA levels in neurons allow for P_tet_bi un-silencing over time. Second, we have introduced an alternative approach for reversible control of gene expression in neurons by using episomal responder delivery via rAAV. This combinatorial genetic approach of combining viruses with genetically altered mice is eminently suitable for targeting cell type specific gene expression in select brain subregions. For example, in combination with *in vivo* 2-photon imaging [23], [39], it is likely to facilitate direct visualization of functional neuronal circuits, by employing genetically-encoded fluorescent biosensors as reporters of cell physiology such as for calcium [40], [41] and synaptic transmission [42].

The defined transcriptional elements of the Tet systems described here should be useful to probe mechanistic issues in regard to chromatin-remodeling in the mammalian neurons and help in identifying factors that modulate promoter silencing and un-silencing in neurons.

## Materials and Methods

### Generation of transgenic mice

Novel synthetic rtTA variants, rtTA2-M2, and, in addition, rtTA2-nM2, containing a simian virus 40 (SV40) 13 amino acid nuclear localization signal (nls) with optimal codons, lacking putative splice acceptor/donor sites [28] and equipped with minimal activation domains [36] were used to produce transgenic mice. Coding sequences for transactivators (rtTA2-M2 and rtTA2-nM2) and the SV40 polyadenylation signal were placed under the αCaMKII promoter fragment [9] resulting in two constructs: αCaMKII-rtTA2-M2 and αCaMKII-rtTA2-nM2. Purified DNA fragments devoid of vector backbone were injected into mouse pronuclei to generate transgenic mouse lines in the facility of Zentrum fuer Molekulare Biologie at the University of Heidelberg. Newly generated mouse lines for each rtTA variant subtype, rtTA2-M2 and rTA2-nM2, have been cataloged as rTA-M2^CaMK^-2, rTA-nM2^CaMK^-4 rTA-nM2^CaMK^-6 rTA-nM2^CaMK^-7 and rTA-nM2^CaMK^-8. In this study, we have also used the forebrain-specific αCaMKII driven tTA expressing mouse lines; the tTA^CaMK^ (line B) [9] and the tTA^CN12^ [29]. Different responder mouse under control of P_tet_bi are all indicated here first by the line name and the two genes in parentheses; LC-1 (luciferase and Cre recombinase) [14], [21], G3 (GFP and lacZ) [22], MTH-Cg2-7 (camgaroo-2 and firefly luciferase), MTH-Cg2-19 (camgaroo-2 and firefly luciferase) and MTH-IP-1 (inverse pericam and firefly luciferase) [23]. All procedures were performed with the German federal guidelines for animal experiments (Licence no. 35-9185.8116-4102, Tubingen, Germany).

### In situ RNA hybridization

*In situ* hybridization studies using ^35^S-labeled oligonucleotide probes were performed according to the method described previously [43]. Saggital 10 µm thick frozen brain slices were prepared by cryostat sectioning from individual animals derived from five different independent rtTA mouse lines. Three oligonucleotide sequences from different coding regions of synthetic tTA/rtTA were hybridized to different brain slices in parallel. All probes gave similar results. The oligonucleotide sequences used are indicated below. Data shown in Fig. 1C-middle and Fig. S1A is from oligo#2.

1. tTA/rtTA-Oligo#1: 5′-TTTAGCTGTTTCTCCAGGCCACATATGATTAGTTCC-3′
2. tTA/rtTA-Oligo#2: 5′-AGCTGATTTTCCAGGGTTTCGTACTGTTTCTCTGTT-3′
3. tTA/rtTA-Oligo#3: 5′-ATAGAATCGGTGGTAGGTGTCTCTCTTTCCTCTTTT-3′

To assess for specificity and rule out non-specific labeling of brain sections, each radiolabeled oligonucleotide was hybridized with a 100-fold excess of unlabelled oligonucleotide. Images were processed after 4 week exposure using Biomax MR (Kodak) X-ray films.

### Routes of Dox delivery

Animals were singly housed in a cage in order to avoid competition for water and food intake. Doxycycline (4-[Dimethylamino]-1,4,4α,5,5a,6,11,12α-octahydro-3,5,10,12,12α-pentahydroxy-6-methyl-1,11-dioxo-2-naphthacenecarboxamide; Sigma-Aldrich, St. Louis, Missouri, United States) was administered to animals by three different delivery routes; drinking water (2 mg per milliliter plus 5% sucrose, fresh every 3 days), food (20 mg per gram plus 20% sucrose, fresh every 3 days) and intraperitoneal injection (i.p) (Dox; 4 mg/300 µL, 9TB-Dox; 1.5 mg per 300 µL). Protocol 1: 9TB-Dox injected every 12 hours. Protocol 2: 9TB-Dox injected every other day. Experiments were performed according to animal guidelines (Licence no. 35-9185.82/A-49106 Karlsruhe, Germany).

### Quantifying gene activity and expression patterns

Mouse brains were saggitally cut into two halves. One half was fixed in 4% paraformaldehyde, the other half was used for the measurement of luciferase activity in different brain subregions. Fixed brain slices were cut to a thickness of 75–100 µm using vibratome (VT 1000S; Leica Instruments, Wetzlar, Germany). Luciferase activity was measured from brain extracts as described previously [6], [21] and immunohistochemistry for Cre recombinase and β-galactosidase was performed also as described previously [14], [21]. Green fluorescence protein (GFP) [44] was visualized in fixed slices either by live fluorescence imaging or by immunohistochemistry using GFP-specific polyclonal rabbit antibodies (Clontech, Mountain View, California, USA) (Krestel et al. 2001) and the DAB peroxidase system (Vectastain ABC Kit; Vector Laboratories, Burlingame, California, USA) or by direct observation of fluorescence with an upright microscope (Zeiss, Oberkochen, Germany) equipped with GFP filters. Dual labeling of neurons for both tTA and GFP was not possible because we needed to employ antigen-specific polyclonal rabbit antibodies for high sensitivity detection of both tTA and GFP.

### Organotypic hippocampus slices

Approximately 300 µm thick hippocampus organotypic slice cultures from P3-P5 pups were prepared as described [45] (double transgenics, rtTA-M2^CaMK^-2×LC-1, positively identified by genotyping) and were cultured at 7% CO2. Doxycycline treatment (1 µg/ml) was started on the day of slicing and was continued for 4 days. Cultures were then fixed for 15 minutes in 4% paraformaldehyde and stained with an anti-Cre rabbit polyclonal antibody (1∶1000) and a Cy3-labeled secondary antibody (1:250, Dianova, Hamburg, Germany). In Fig. 2B, images are presented in grey scale.

### Adeno-associated viral mediated gene transfer into mouse brain tissues

Recombinant adeno-associated virus (rAAV) [27] equipped with the P_tet_bi [4] and harboring synthetic Cre recombinase (miniCre) [46] and a GFP-variant (Venus) [47] was used to generate the plasmid rAAV-P_tet_bi-GFP/Cre. Plasmids, rAAV-P_tet_bi-GFP/Cre, rAAV-hSyn-tTA and rAAV-hSyn-rtTA2-nM2 were individually co-transfected with pDp1, pDp2 (ratio: 3∶1) helper plasmids [48] in HEK293 cells. Seventy-two hours after transfection, HEK293 cells were collected and packaged viruses were released by repeated freeze-and-thaw on dry-ice-ethanol bath. Viruses were purified by pre-casted 5ml Heparin columns (Amersham, Freiburg, Germany). Infectious virus titers were determined in primary neuron cultures. Viruses were delivered through thin glass pipettes to specific brain sites by stereotaxic injection using the SAS75 stereotaxic alignment system with combination of EM70G manipulator (Kopf Instruments, Germany). Infected animals were kept for 14–21 days before analysis of brain tissues. All experiments were carried out according to the biosafety guidelines specified in the German GenTSV (Regierungprasidium Tubinger, A2:35-9185.8/10-56105).

### Sodium bisulphite conversion, PCR amplification and sequencing

Genomic DNA was prepared according to standard procedures. Sodium bisulphite treatment of genomic DNA was performed as described previously [49] with minor modifications. Since methylated cytosines have the same base-pairing characteristics as unmethylated cytosines, DNA is chemically modified to distinguish between the two species. Purified genomic DNA was treated with sodium bisulphite, resulting in the conversion of unmethylated cytosine to uracil. In a subsequent PCR uracil is replicated as thymine. Methylated cytosines are protected from conversion and remain as cytosines. Thus, detection of a “C” in a sequencing reaction reflects methylation at that site whereas detection of a “T” indicates no methylation. PCRs were performed on MJ Research thermocyclers (Waltham, Massachusetts, United States) in a final volume of 25 µl containing 1× PCR Buffer, 1 U */Taq/* DNA polymerase (Qiagen), 200 µM dNTPs, 12.5 pmol each of forward and reverse primers, and 7 ng of bisulphite-treated genomic DNA. The amplification conditions were 95°C for 15 min and 40 cycles of 95°C for 1 min, 55°C for 45 sec and 72°C for 1 min and a final extension step of 10 min at 72°C. PCR products were purified using ExoSAP-IT (USB Corp., Cleveland, Ohio, United States) and sequenced employing the PCR primers and the ABI Big Dye Terminator v1.1 cycle sequencing chemistry (Applied Biosystems, Foster City, California, United States) followed by capillary electrophoresis on an ABI 3100 genetic analyzer. AB1-files were interpreted using ESME, which normalizes sequence traces, corrects for incomplete bisulphite conversion and allows for quantification of methylation signals. The following primers were used both for PCR amplification of bisulfite converted genomic DNA and sequence reactions:

1. Amp806 fw: 5′-TATAGTTTTATGTAGTTGTTTTTTAG-3′ and
2. Amp806 rev: 5′-AATAAATTAAACACCTTCCTC-3′.

PCR products were cloned using the TOPO TA Cloning Kit (Invitrogen). Sequencing was carried out using the M13 Forward and M13 Reverse primers by 3100 Genetic Analyzer (Applied Biosystem). All bisulphite sequencing experiments were performed in duplicate and sequencing data was analyzed using SeqMan (Lasergene, DNASTAR, USA). The conversion rate was approxinately 95%. We have fully sequenced and characterized 71 clones.

## Supporting Information

Figure S1 Expression of rtTA mRNA and Cre protein in the brain of different mouse lines. (A) Detection of rtTA-M2 mRNA in the brain slices of different rtTA2-M2^CaMK^ mouse lines. Specific signal detected with radiolabeled oligonucleotides for rtTA2-M2 (left) and non-specific signal (right). (B) 9TB-Dox induced, rtTA-dependent Cre expression in different brain regions (rtTA2-M2^CaMK^ lines crossed to LC-1 responders). Abbreviations: Cx (cortex), Hi (hippocampus), Ce (cerebellum), St (striatum) and Ob (olfactory bulb). Scale bars, 2 mm (A) and 1 mm (B).
(6.30 MB TIF)

Figure S2 Forebrain-specific, tTA-dependent gene activation in responder mice MTH-Cg2-17 and MTH-Cg2-19. (A, B) single-positive (without tTA^CaMK^) (left panel) and double-positive (with tTA^CaMK^) (right panel).
(8.22 MB TIF)

Figure S3 9TB-Dox induced, rtTA-dependent gene activation in MTH-Cg2-19. (A) with rtTA-M2^CaMK^-2 and (B) with tTA^CaMK^. Scale bar, 500 µm.
(9.49 MB TIF)

Figure S4 Methylation of the P_tet_bi. (A) The bidirectional tetracycline-responsive promoter (P_tet_bi) is depicted and CpG sites are indicated as open circles. (B) Sequence analysis of 71 individual clones is plotted with the number of methylated CpG detected (y-axis) against the number of independent clones (x-axis). The actual data set collected is based on methylation detected from sequencing (red) and the calculated data is based on 5% C-to-T conversion loss (blue). Three of seventy-one clones (4%) are strongly methylated.
(0.47 MB TIF)

Figure S5 Notes on P_tet_bi methylation analyses.
(0.02 MB DOC)
