# Supplementary figures and images for: Silencing and Un-silencing of Tetracycline-Controlled Genes in Neurons
Source: PLoS One. 2007 Jun 20;2(6):e533. doi: 10.1371/journal.pone.0000533 (PMC1888723; doi:10.1371/journal.pone.0000533)

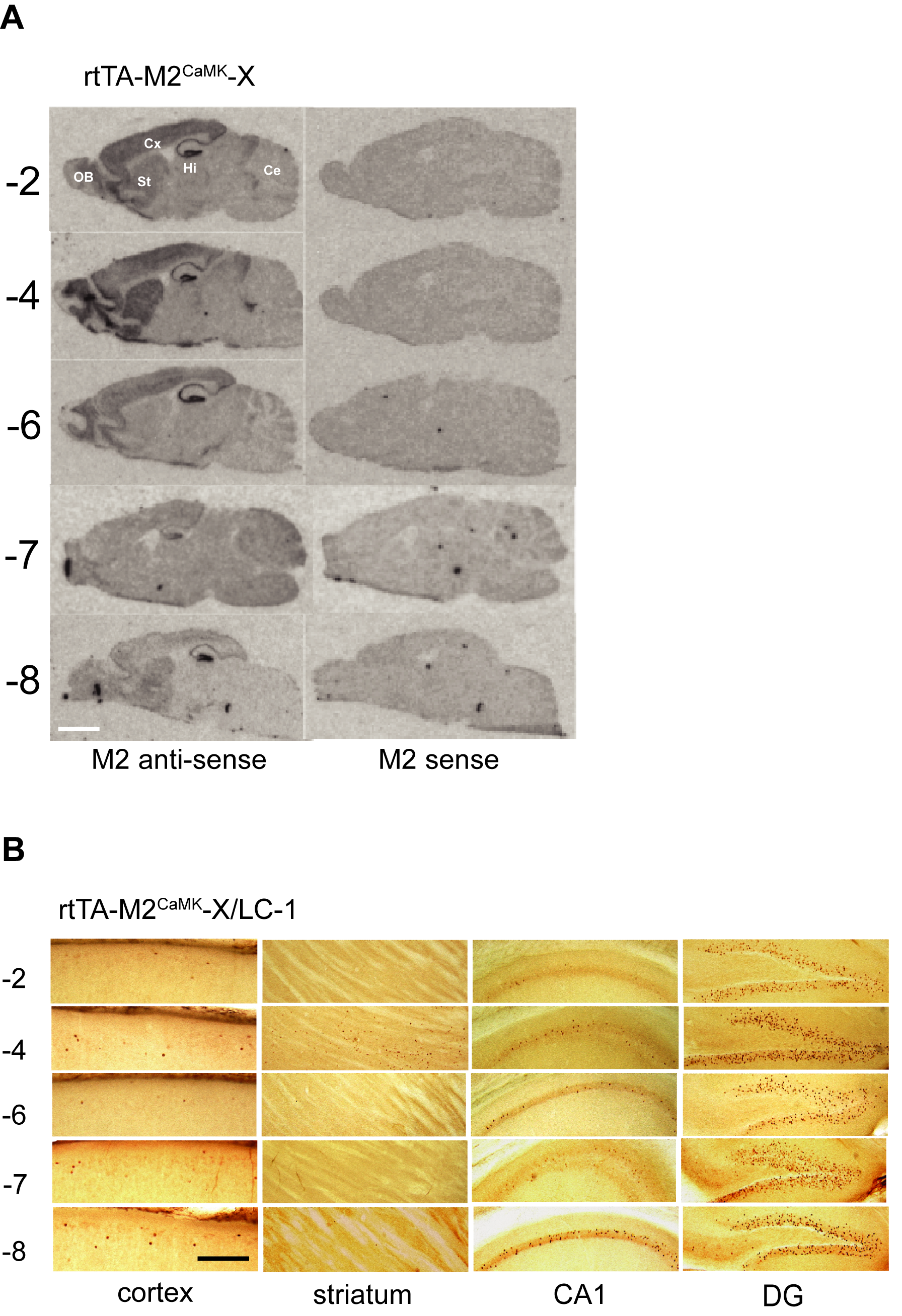

Supplement: Figure S1 — Expression of rtTA mRNA and Cre protein in the brain of different mouse lines. (A) Detection of rtTA-M2 mRNA in the brain slices of different rtTA2-M2CaMK mouse lines. Specific signal detected with radiolabeled oligonucleotides for rtTA2-M2 (left) and non-specific signal (right). (B) 9TB-Dox induced, rtTA-dependent Cre expression in different brain regions (rtTA2-M2CaMK lines crossed to LC-1 responders). Abbreviations: Cx (cortex), Hi (hippocampus), Ce (cerebellum), St (striatum) and Ob (olfactory bulb). Scale bars, 2 mm (A) and 1 mm (B). (6.30 MB TIF) [file pone.0000533.s001.tif]

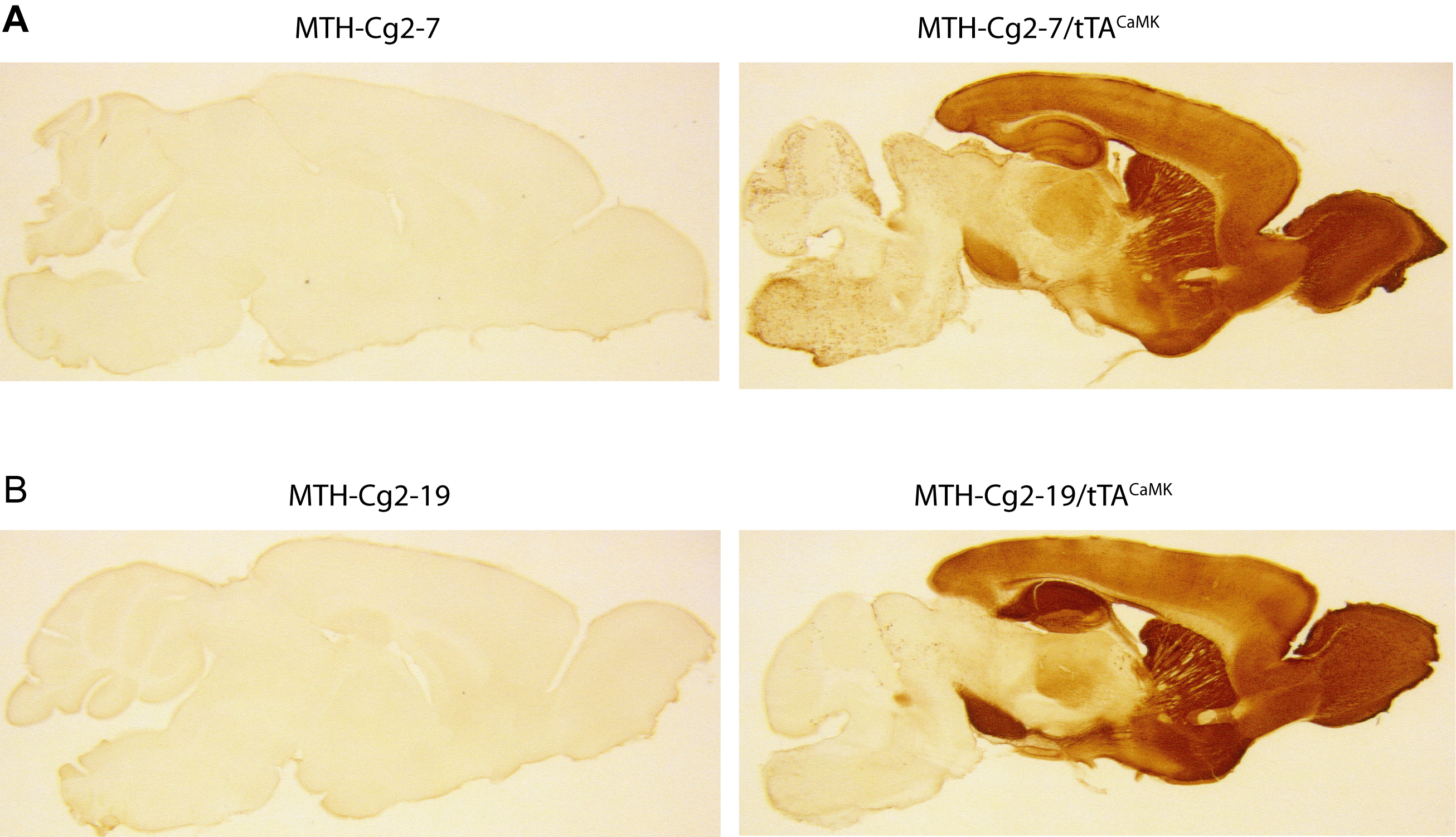

Supplement: Figure S2 — Forebrain-specific, tTA-dependent gene activation in responder mice MTH-Cg2-17 and MTH-Cg2-19. (A, B) single-positive (without tTACaMK) (left panel) and double-positive (with tTACaMK) (right panel). (8.22 MB TIF) [file pone.0000533.s002.tif]

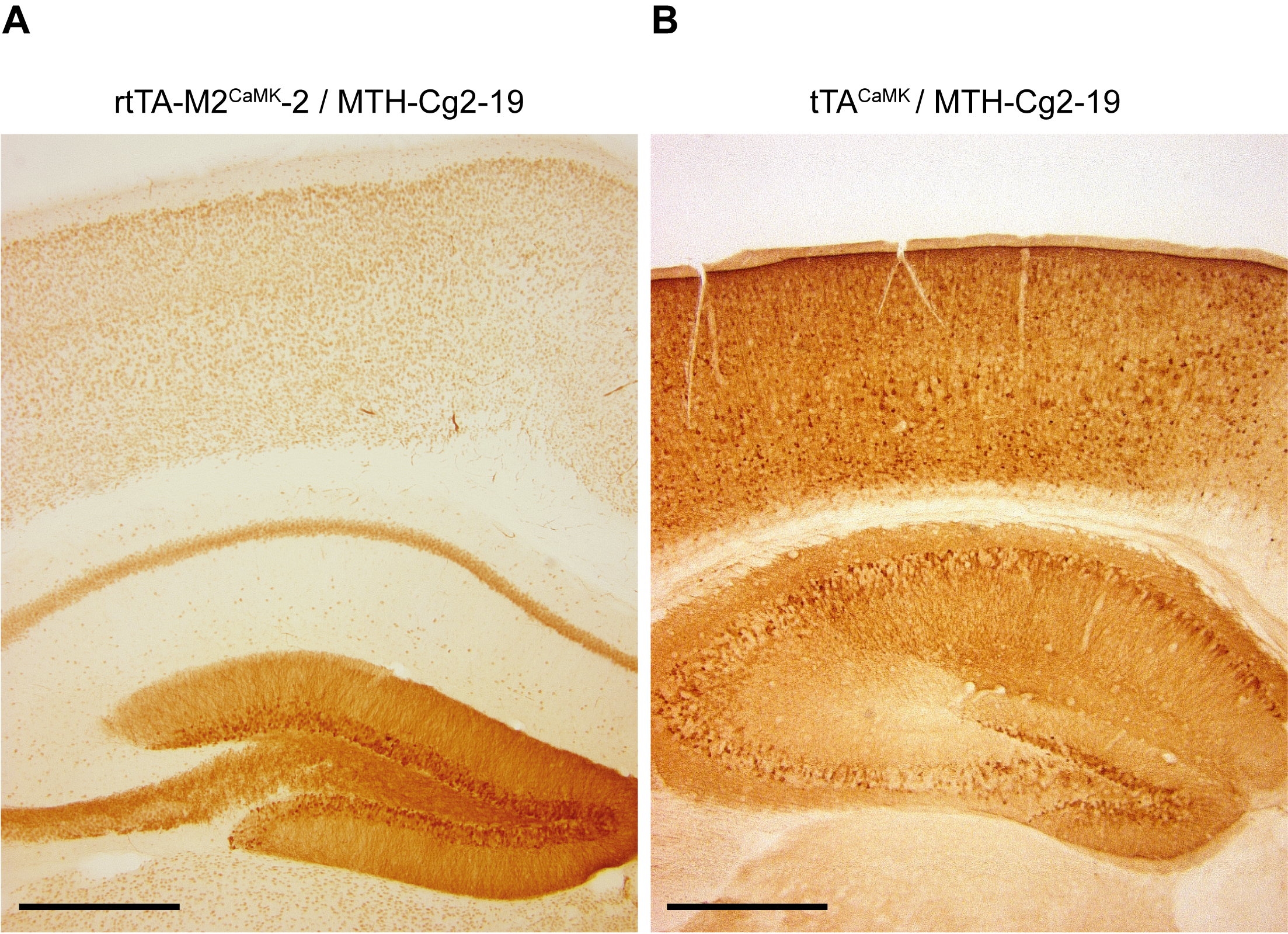

Supplement: Figure S3 — 9TB-Dox induced, rtTA-dependent gene activation in MTH-Cg2-19. (A) with rtTA-M2CaMK-2 and (B) with tTACaMK. Scale bar, 500 µm. (9.49 MB TIF) [file pone.0000533.s003.tif]

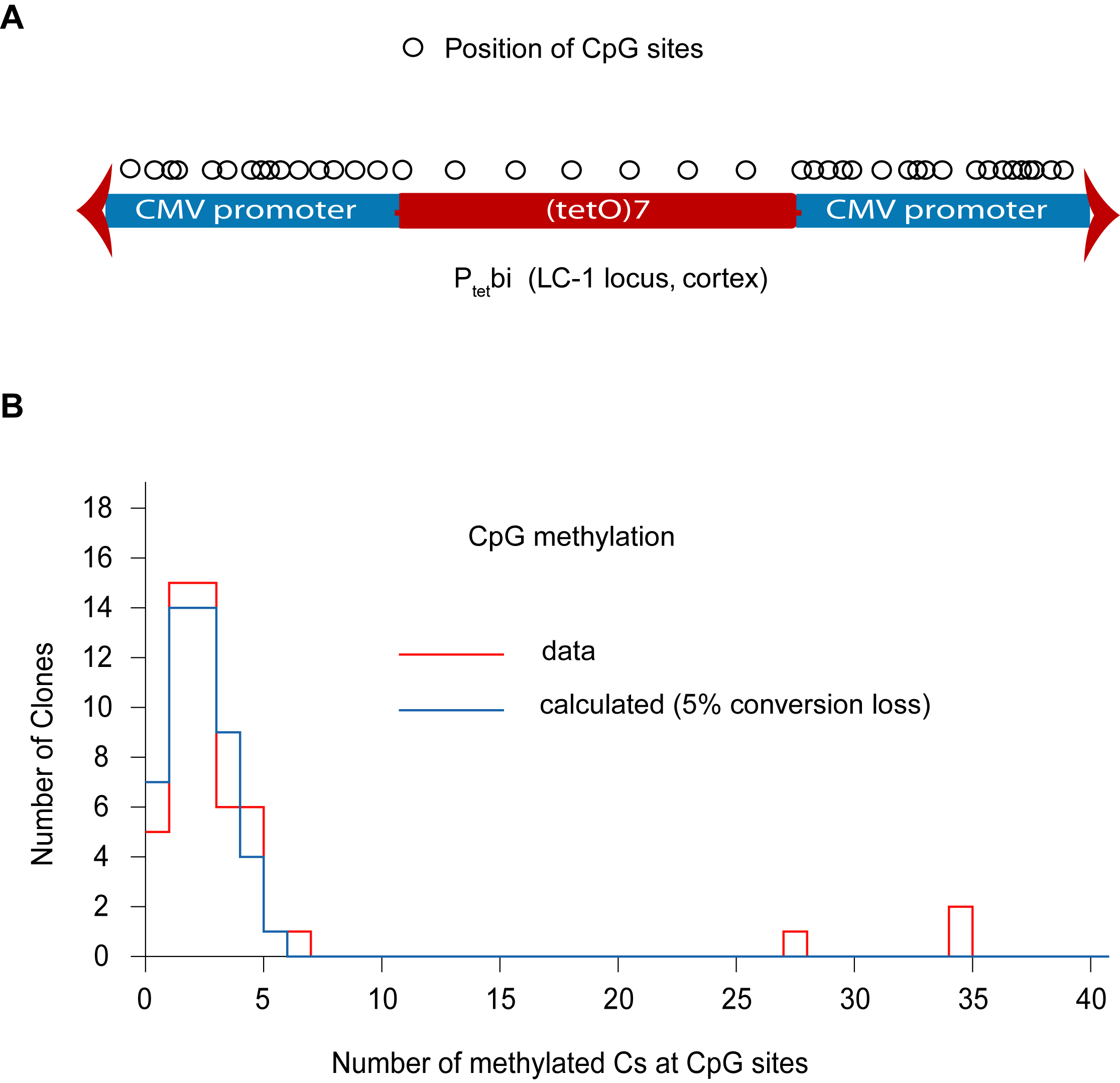

Supplement: Figure S4 — Methylation of the Ptetbi. (A) The bidirectional tetracycline-responsive promoter (Ptetbi) is depicted and CpG sites are indicated as open circles. (B) Sequence analysis of 71 individual clones is plotted with the number of methylated CpG detected (y-axis) against the number of independent clones (x-axis). The actual data set collected is based on methylation detected from sequencing (red) and the calculated data is based on 5% C-to-T conversion loss (blue). Three of seventy-one clones (4%) are strongly methylated. (0.47 MB TIF) [file pone.0000533.s004.tif]
