# Supplementary material for: Silencing and Un-silencing of Tetracycline-Controlled Genes in Neurons
Source: PLoS One. 2007 Jun 20;2(6):e533. doi: 10.1371/journal.pone.0000533 (PMC1888723; doi:10.1371/journal.pone.0000533)
Supplement: Figure S5 — Notes on Ptetbi methylation analyses. (0.02 MB DOC) [file pone.0000533.s005.doc]

**Supplementary Material 5**

Notes on P_tet_bi methylation

Lack of rtTA-dependent gene activation in a majority of neurons prompted us to examine the DNA methylation in P_tet_bi from genomic DNA derived from the cortex of adult LC-1 responder mice which were developmentally inactive. The methylation patterns of the LC-1 locus from cortical genomic DNA were analyzed by bisulphite sequencing (Olek et al. 1996) applying amplicons 806 spanning the tetracycline inducible bidirectional promoter region (Supplementary Material 4A). While the CMV promoter region (“the small one”) driving luciferase (CpGs 118-210) was found to be methylated to an intermediate level (~30%), the other CMV promoter region (“the large one”) driving cre recombinase (CpGs 524-734) was methylated to a much lower level. The heptameric tetO region (CpGs 230-554) was shown to be unmethylated. Since CpG methylation rates were measured from the entire cortex composed of multiple cell types, we are not certain that methylation signals originate from particular neuronal cell types in an otherwise unmethylated background which might also include a variety of non-neuronal cell types, including microglia, astrocytes and blood vessel cells. It is known that the entire cortex composed of multiple cell types of which about 60% are usually neurons (per cubic millimeter) in mice (Bass et al. 1971).

To get a better idea on patterns of DNA methylation in individual cells from this mixture, we generated a plasmid library of bisulphate-converted PCR-generated products and sequenced 71 individual clones. Because there is incomplete conversion (about 95%, data not shown), we compared the predicted loss of C-to-T conversion to the actual data set from sequence analyses to eliminate false-positive (Supplementary Material 4B). Our results show that CpGs are strongly methylated in a small pool (3/71 clones, ~4%) and is distributed throughout the entire P_tet_bi. Based on the incomplete conversion, which is assumed to be a independent random process, the probability (binomial distribution) to find a single clone with 34 unconverted Cs is $2.6\times10^{-39}$. Therefore the three strongly methylated clones most likely represent real events from DNA in the mouse cortex.
